# Calcium Imaging and Electrophysiology of hippocampal Activity under Anesthesia and natural Sleep in Mice

**DOI:** 10.1038/s41597-022-01244-2

**Published:** 2022-03-29

**Authors:** Andrey Formozov, Mattia Chini, Alexander Dieter, Wei Yang, Jastyn A. Pöpplau, Ileana L. Hanganu-Opatz, J. Simon Wiegert

**Affiliations:** 1 Research Group Synaptic Wiring and Information Processing, Center for Molecular Neurobiology Hamburg, University Medical Center Hamburg-Eppendorf, 20251 Hamburg, Germany; 2 Institute of Developmental Neurophysiology, Center for Molecular Neurobiology Hamburg, University Medical Center Hamburg-Eppendorf, 20251 Hamburg, Germany

**Keywords:** Neuronal physiology, Dynamical systems, Fluorescence imaging, Extracellular recording

## Abstract

The acute effects of anesthesia and their underlying mechanisms are still not fully understood. Thus, comprehensive analysis and efficient generalization require their description in various brain regions. Here we describe a large-scale, annotated collection of 2-photon calcium imaging data and multi-electrode, extracellular electrophysiological recordings in CA1 of the murine hippocampus under three distinct anesthetics (Isoflurane, Ketamine/Xylazine and Medetomidine/Midazolam/Fentanyl), during natural sleep, and wakefulness. We cover several aspects of data quality standardization and provide a set of tools for autonomous validation, along with analysis workflows for reuse and data exploration. The datasets described here capture various aspects of neural activity in hundreds of pyramidal cells at single cell resolution. In addition to relevance for basic biological research, the dataset may find utility in computational neuroscience as a benchmark for models of anesthesia and sleep.

## Abstract (summary)

**Table:** 

Measurement(s)	brain activity measurement • pupil dilation
Technology Type(s)	two-photon calcium imaging • local field potential recording • videography
Factor Type(s)	calcium imaging data • local field potentials
Sample Characteristic - Organism	Mus musculus

## Background & Summary

Anesthesia is one of the most frequently applied procedures both in basic research and in the clinic. Still, the effects of anesthesia on neuronal activity, subsequent recovery, and potential long-term brain function alterations are poorly understood. The relationships as well as the differences between artificially induced unconscious states and natural sleep are also of great interest, and knowledge in this respect does not yet have a common theoretical and empirical basis. The lack of large-scale datasets hinders the detection of subtle effects on the population and single-neuron level. Effective generalizations will require high-quality annotated datasets recorded from various brain regions, at different time-scales, and using several experimental techniques. We recently published a study which systematically investigated the functional and structural properties of CA1 in the dorsal hippocampus under three different anesthetics: Isoflurane (Iso), Ketamine/Xylazine (Keta/Xyl), and Medetomidine/Midazolam/Fentanyl (MMF)^1^. We further investigated recovery from anesthesia, and compared the effects of these anesthetics to wakefulness and sleep.

The present document describes three main datasets generated during this study, capturing the neural correlates of the different physiological states by two-photon imaging with the genetically encoded calcium indicator GCaMP6f ^2^ and by extracellular electrophysiological recordings, in different experimental settings. We further provide video recordings of the animal’s eye during sleep and wakefulness. In addition to the raw data recordings, we also describe the resulting pre-processed files to enhance overall reusability.

Guided by established principles of data validation^3^, we developed several procedures for data quality control based on recent advances in calcium signal extraction algorithms^4^. In addition to the data and validation scripts, we provide Jupyter notebooks linked to the Google Colab platform to further increase usability. The notebooks cover essential steps of the data validation procedure such as controlling for quality, stability, and homogeneity of the recordings. In addition, we link two GitHub repositories, which provide code and raw/processed data to replicate the results of Yang *et al*.^1^ and describe various strategies to reuse the data. The Data Descriptor may be useful in computational neuroscience, where benchmarking of simulations against biological data is of high importance (see e.g.^5^). Moreover, our systematic comparison of different anesthetic and physiological conditions using two different recording techniques (electrophysiology and calcium imaging) facilitates the exploration of their advantages and drawbacks^6^. Finally, the provided infrastructure has an additional value covering a broad spectrum of tools for the analysis of imaging and electrophysiological data.

## Methods

Details on the experimental procedures, including data collection, are described in the original publication^1^. Below, we provide a description of the experimental design together with specific information tailored to the technical validation and proper reuse of the data.

### Experimental design

**Fig. 1:**
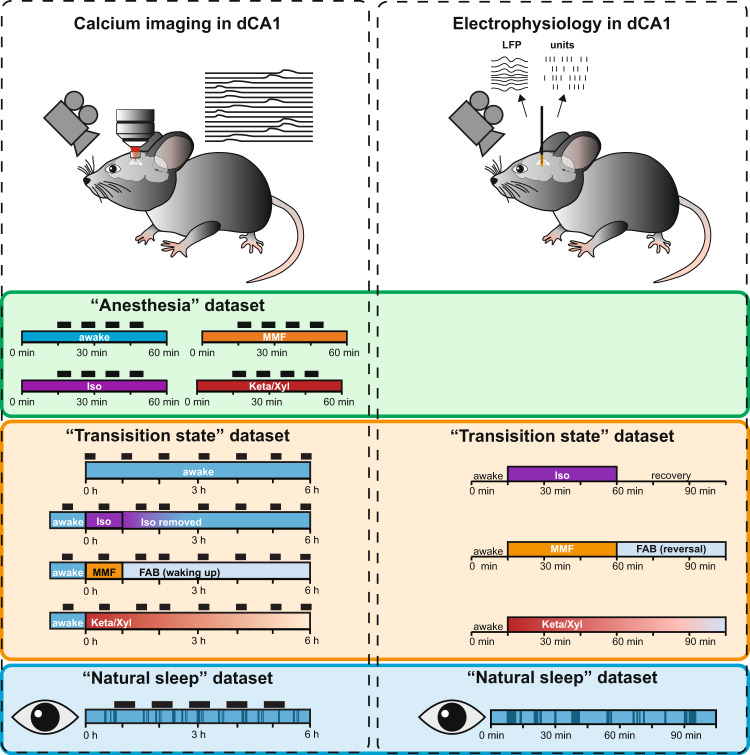
Experimental design and data-acquisition procedures for two-photon calcium imaging (left) and electrophysiological recordings (right) from the mouse hippocampus. The calcium imaging “Anesthesia” dataset was acquired in blocks of 5 min (indicated by black rectangles) in each condition (awake, isoflurane, MMF, and Keta/Xyl). The “Transition State” and “Natural Sleep” datasets capture 6–6.5 h of monitoring with blocks of calcium recordings lasting from 2 to 19 min, each. Electrophysiological recordings were acquired continuously for 105 min (“Transition State”) or for 67 to 147 min (“Natural Sleep”). Pupillometry recordings accompany all “Natural Sleep” datasets.

The datasets described here monitor CA1 activity by calcium imaging and electrophysiology in the following experimental settings (Fig. 1): the main calcium imaging dataset collected to assess the different anesthetics consists of multiple five-minute recordings in awake or anesthetized conditions (Fig. 1, “Anesthesia” dataset). The dataset includes imaging sessions with animals in four distinct conditions: under the administration of Iso, Keta/Xyl, or MMF, or during quiet wakefulness.

To capture the modulation of neuronal activity before, during, and after anesthesia, two additional calcium imaging and electrophysiology datasets were acquired (Fig. 1, “Transition State” dataset). The imaging of calcium activity was performed before, 0.5, 1.5, 2, 3, 4, 5, and 6 h after induction of anesthesia. Iso was delivered via inhalation for 60 min. A mix of Flumazenil/Atipamezole/Buprenorphine (FAB) was injected 60 min after the injection of MMF to antagonize anesthesia. Keta/Xyl was also administered via intraperitoneal injection, but not antagonized. For Iso and MMF, the 1.5-hour time point represented the first imaging session after reversal. Untreated control animals during quiet wakefulness were imaged every hour instead.

In the electrophysiology dataset, animals were recorded for 15 min of baseline, followed by a brief interruption to administer anesthesia: a subcutaneous injection of MMF or Keta/Xyl, or the inhalation of Isoflurane. Following anesthesia induction, extracellular recordings were uninterruptedly recorded for 45 min before anesthesia was terminated: mask removal in the case of Isoflurane and injection of the antagonization cocktail in the case of MMF. No antagonization was applied to Keta/Xyl. After a brief interruption, further 45 min of recovery were recorded.

In the third experimental setting, animals were habituated to head-fixed 2-photon imaging or electrophysiological recordings during quiet wakefulness and different sleep states (Fig. 1, “Natural Sleep” dataset). The durations of recordings were kept the same as in the “Transition State” dataset. Electrophysiological recordings in the sleep-state setting were accompanied by neck muscle electro-myogram (EMG) to distinguish between sleep- and awake-states. In both sets of experiments, video-recordings of the eye were performed in parallel. Using LFP and EMG recordings of the electrophysiology dataset as ground truth, sleep-states in the calcium imaging dataset could then be extracted from pupil- and eyelid dynamics as previously described^1,7^. Briefly, sleep states were initially classified with a rule-based approach. This classification was then extended using a supervised machine-learning algorithm that reduced the number of unclassified epochs. Importantly, we focused on consolidated behavioral epochs and, even after the second classification step, ~28% of the epochs were left unclassified. Users who prefer to optimize other criteria might want to carry out a different sleep classification using the EMG/LFP data provided in the repositories.

### Animals

To generate the datasets, adult (3 to 9 months of age) C57BL/6 J mice of both sexes were used. The light/dark cycle during housing was 12/12 h; humidity and temperature were kept constant (~40% relative humidity; ~22 °C). Food and water were available ad libitum. All procedures were performed in compliance with German law and in accordance with the guidelines of the Directive 2010/63/EU. Protocols were approved by the Behörde für Justiz und Verbraucherschutz of the City of Hamburg.

### Calcium imaging

Adeno-associated virus (serotype rAAV2/7) carrying plasmids encoding for GCaMP6f under control of the human synapsin promoter (hSyn-GCaMP6f) was targeted unilaterally to the dorsal CA1 area (0.6 µl of viral suspension; 2.0 mm posterior, ±1.3 mm lateral, −1.5 mm ventral relative to Bregma). A custom hippocampal window was implanted two weeks later above the injection site. During this procedure, the dura mater and cerebral cortex above the hippocampus were carefully removed. For awake states in the “Anesthesia” and “Natural Sleep” datasets, head-fixed mice were placed on a linear treadmill. Anesthetized mice were instead located on a heated blanket to maintain body temperature at approximately 37 °C. Prior to the “Natural sleep” dataset acquisition, animals were habituated for 2-3 weeks to sleep in the experimental environment. All experimental settings for 2-photon imaging except the power of the excitation laser were kept fixed across all imaging sessions: Ti:Sa laser (Chameleon Vision-S, Coherent) tuned to 980 nm, 16x water immersion objective (Nikon CFI75 LWD 16X W, 0.80 NA, 3.0 mm WD), 512 × 512 pixels per frame with 30 frames per second imaging rate. Excitation power was adjusted in the range of 9–132 mW to achieve visually comparable fluorescence of GCaMP6f-expressing pyramidal cells across animals (see Technical Validation section for details). The field of view (FOV) across different imaging sessions was kept the same for each animal by using the vascular landmarks and cell bodies for FOV-alignment. In addition to that, for the “Transition State” and “Natural Sleep” data, the alignment tool provided with ScanImage 2017b^8^ was used between the recording sessions to improve the field of view homogeneity. The recordings were acquired in pseudo-randomized order with respect to the treatment; blinding was not employed. The following quality criteria were applied: 1) absence of hippocampal damage and normal motor behavior after cranial window implantation, which requires partial removal of the neocortex. 2) sufficient GCaMP6f-signal in CA1 during 2-photon imaging (on the latter, see validation section for more details). 1 of 15 animals was excluded due to insufficient GCaMP6f expression after the first imaging session; and the hippocampal window for one more animal (ID M0) was damaged after two recording sessions (for the remaining sessions the animal was replaced with mouse ID M3). The raw calcium data are available in^9^. To conduct validation procedure and to facilitate efficient reuse of the recordings, raw data were pre-processed with the Suite2p pipeline^4^. Pre-processed data are available in^10^.

### Electrophysiological recordings

Electrophysiological recordings were acquired with a silicon probe (NeuroNexus, Michigan, United States of America - one-shank, A1x16 recording sites, 50-μm spacing) inserted in the dorsal CA1 to a depth of 1.6 mm. The signal was acquired with an extracellular amplifier (Digital Lynx SX; Neuralynx, Bozeman, Montana, USA) at 32 kHz sampling rate and with a band-pass filter in the 0.1–8000 Hz range. No animal was excluded to produce the datasets. The electrophysiological datasets for anesthesia and sleep are available in “electrophysiology” and “Sleep/LFP” folders in^10^.

### Pupillometry

Video-graphic recordings of the animal’s eye accompanied both 2-photon calcium imaging and extracellular electrophysiological recordings in the “Natural Sleep” datasets. Macro-objective equipped, monochrome cameras (UI-3360CP-NIR-GL Rev. 2, [iDS imaging] & LMZ45T3 2/3” 18 to 108 mm/F2.5 zoom lens [Kowa] for electrophysiology and DMK 33UX249 [The Imaging Source] with a TMN 1.0/50 objective [The Imaging Source] for calcium imaging) were directed at the animal’s eye, and videos were obtained in parallel to neuronal recordings at frame rates of 30 and 10 Hz, respectively. Electrophysiological experiments were performed under darkened room light. During calcium imaging, the animal was placed in darkness and the eye was illuminated with an 810 nm infrared-LED, while the camera objective was equipped with an 810/27-nm bandpass filter. Subsequently, features of the eye (upper, lower, left and right edge of the eyelid and the pupil, respectively) were extracted from video-graphic images using the deep neural network-based software module DeepLabCut^11^. To reduce computation times, videos were previously down-sampled to a size of 256 pixels on the shorter edge. Samples in which the feature detection had a low certainty (<0.5, as defined by DeepLabCut) were linearly interpolated, and the pupil diameter (maximum distance between left/right and top/bottom edge of the pupil) and eyelid opening (distance between top/bottom of the eyelid) were then calculated. Eyelid blinks (defined as periods not exceeding a threshold of a moving median [sliding window of 30 sec] minus 3 moving median absolute deviations, as blinks are always decreasing the eye opening) were also removed from the eye-opening data by linear interpolation. Pupillometry recordings accompanying “Natural sleep” datasets are available in the “Sleep_pupillometry” folder of the dataset^9^ and in “Sleep/pupillometry_ephys” folder of the pre-processed dataset^10^.

## Data Records

### Calcium imaging datasets

The calcium imaging dataset consists of three distinct sub-datasets: “Anesthesia” (189 recordings, 5 min fixed), “Transition State” (172 recordings: awake, anesthesia and subsequent recovery from anesthesia, 2.8–18.9 min), and “Natural Sleep” (54 recordings, 2.1–14.8 min duration). Raw data are stored as *.tif* files under the directories with the date of the imaging session and mouse identification number^9^; the full list of files is available in corresponding meta-data files (*.xlsx or *.csv), in the “meta_data” folder. Preprocessed data (calcium transients) are available in^10^. The recordings are listed in Table 1, along with animal IDs, condition (awake, sleep, anesthesia), number of recordings, recording duration in minutes. Two strategies were considered for pre-processing of the calcium data: extraction of calcium traces per recording (“Anesthesia” and “Sleep” datasets) and extraction from concatenated recordings after their alignment (“Transition State” dataset). In the latter case, all recordings in awake, anesthesia, and recovery conditions were aligned and merged into a single file, preserving the identity of neurons between recordings. One may refer to the meta-data file to split concatenated traces into single-recording traces by selecting the corresponding range of frames. Table 2.

**Table 1:** Two-photon calcium imaging data: “Anesthesia”, “Transition State”, and “Natural Sleep” datasets^9^.

Mouse ID	Condition	Recordings (no.)	Duration (min.)
**“Anesthesia” dataset**			
37527, 37528, 37529, 37530, 48, 51, 53	Keta/Xyl	50	5.0
	Isoflurane	49	5.0
	MMF	51	5.0
	Awake	39	5.0 (10.0 in some recordings)
**“Transition State” dataset**			
8235, 8237, 8238, F0, M0 (M3), F1	Awake - Anesthesia - Recovery	162	2.8–18.9
	Awake (control)	37	4.2–11.0
**“Natural Sleep” dataset**			
8235, 8237, 8238	Natural sleep - awake	54	2.1–14.8

**Table 2:** LFP recordings with corresponding SUA data: “Natural Sleep” and “Transition State” datasets.

Mouse ID	Condition	Recordings (no.)	Duration (h.)
**“Transition State” dataset**			
1407	Keta/Xyl	1	1.5
	Isoflurane	1	
	MMF	1	
1408	Keta/Xyl	1	
	Isoflurane	1	
	MMF	1	
47511	Keta/Xyl	2	
	Isoflurane	2	
	MMF	2	
**“Natural Sleep” dataset**			
M1	Natural sleep-awake	2	1.5
M2		2	
M3		1	
M4		2	

### Electrophysiology datasets

LFP data were down-sampled to 320 Hz and band-pass filtered using a third-order Butterworth forward and backward filter in the 0.1–160 Hz frequency range. Only the “reversal” channel (putatively corresponding to signal coming from the CA1 stratum pyramidale) is provided. Single Unit Activity (SUA) data has been clustered using *klusta* and manually curated in the *phy* environment. For every SUA recording, we provide a spike matrix, which is a binary matrix with unit ID corresponding to the first dimension and spike time (in 1 ms bins) to the second dimension. The spike matrices have been divided in three subparts corresponding to “baseline”, “anesthesia” and “recovery”. For each of the three spike matrices, we further provide a “SUAinfo” file, which gives the following information for every single unit:

- the channel from which the unit was recorded
- the timestamps at which the unit fired an action potential
- its cluster ID
- its median waveform
- the amplitude of every action potential
- the number of refractory period violations (the number of action potentials that occurred less than 2 ms apart from each other)
- 4 features of its waveform: half-width, amplitude, trough to peak, and an asymmetry index

All the electrophysiology data (LFP and SUA) is saved in the *.mat format and available in^10^, under “Anesthesia/electrophysiology” (“Transition State” dataset) and under “Sleep” (“Natural Sleep” dataset). The recordings are listed in Table 2.

### Pupillometry datasets

Videos of the animal’s eye were recorded to classify awake and sleep states, and hence are only available for the calcium imaging and electrophysiology “Natural Sleep” datasets in “Sleep_pupillometry/raw_2PM” and “Sleep/pupillometry_ephys” folders of^9^ and^10^ datasets, respectively. For these recordings, we provide raw videos (.*mp4*), DeepLabCut-extracted feature positions indicating an X- and Y-coordinate along with a certainty value of feature detection for each feature, respectively (.*csv*), as well as raw and pre-processed pupil diameter and eyelid opening values of all pupil recordings (*.mat*). Basic features of the dataset acquired during calcium imaging versus the dataset acquired during electrophysiological recordings are summarized in Table 3.

**Table 3:** Basic parameters of pupil recordings in sleep datasets.

	Calcium imaging	Electrophysiology
Camera	DMK 33UX249	UI-3360CP-NIR-GL Rev. 2
Objective	TMN 1.0/50	LMZ45T3 2/3” 18 to 108 mm/F2.5
Filter	810/27 bandpass	—
Illumination	IR illumination (810 nm)	darkened room light
Frame rate	10 Hz	30 Hz
Resolution	256 × 320	256 × 274
# animals	3	4
# sessions	54	7
Session duration (min)	6.6 ± 3.27	322.17 ± 71.2
Total duration (min)	356	2255

## Technical Validation

The technical validation of the calcium imaging datasets beyond visual inspection requires a broad analysis of the quality and stability of the extracted signals^3^. To address this issue, we developed a validation routine consisting of (1) pre-processing the calcium data with the Suite2p pipeline^4^ and subsequent signal feature extraction, (2) analysis of the homogeneity and stability of features across datasets, (3) signal-to-noise ratio estimation, (4) analysis of FOV alignment and (5) motion artefacts. Electrophysiology datasets were validated in terms of power spectral density, refractory period violations (after extraction of SUA), and stability of firing rates in the awake condition. The verification of the quality and variability of pupillometry recordings was conducted with the analysis of the homogeneity of the features in sleep and awake periods extracted by a machine learning-based algorithm^11^.

### Stability of recordings and signal-to-noise ratio

**Fig. 2:**
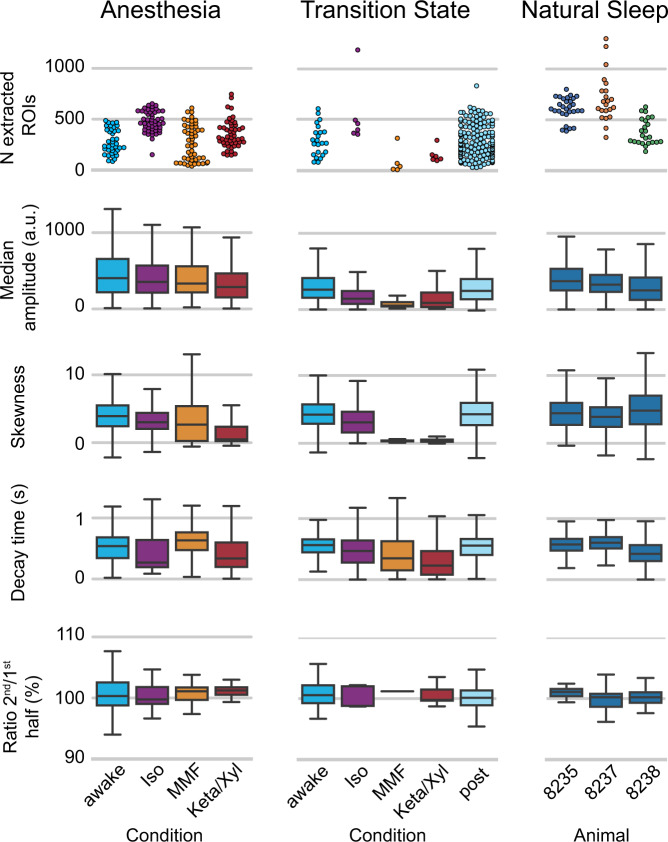
Technical validation of the calcium imaging datasets. Several basic characteristics of the recordings are considered for validation. Number of extracted ROIs (one data point per recording), distribution of the median amplitude and skewness of the signal traces for all ROI, decay time of transients extracted from the traces, and 2^nd^/1^st^ ratio (the ratio of the median signal of the first and the second half of the traces in each recording) as an indicator of recording stability. The box plots represent the distribution of a parameter in the range between the 1^st^ (Q1) and 3^rd^ (Q3) quartile, the central line is a median (2^nd^ quartile, Q2), while whiskers defined as 1.5 * (Q3 – Q1). Recordings in “Anesthesia” (n = 7 animals) and “Transition State” (n = 7) datasets are grouped by condition, while in “Natural sleep” (n = 3) dataset, they are grouped by animal.

To ensure homogeneity across the hundreds of recordings, we developed a validation routine that compares the number of detected neurons (both accepted and rejected by the classifier), the median signal, the skewness of individual traces, and local recording stability of these recordings. The calcium imaging datasets were pre-processed with the Suite2p pipeline for trace extraction^4^. The number of extracted ROIs classified as neurons is generally consistent across conditions, ranging for awake/anesthetized condition between 160 and 635 ROIs per recording in the “Anesthesia” dataset, between 110 and 630 in the “Transition State” dataset, and between 290 and 1220 in the “Natural sleep” dataset (95% C.I.). Recordings under MMF and Keta/Xyl conditions in the “Transition State” dataset generally have less detected ROIs compared to the main “Anesthesia” dataset as they were recorded in the deepest phase of anesthesia when neuronal activity was minimal (~30 min after administration of MMF or Keta/Xyl). The dataset was acquired with fine adjustments of the laser excitation power to keep the distribution of the fluorescence intensities comparable between imaging sessions, which resulted in consistent values of the median signals (Fig. 2, *Median*) regardless of the expression levels of the indicator.

We used skewness of the trace distribution for a given ROI as a proxy for the magnitude of neuronal responses. Skewness was consistently ≈ 5 in all recordings across awake, sleep and isoflurane conditions. While for the two other anesthesia conditions (Keta/Xyl and MMF), the significant reduction of neuronal activity led to much smaller skewness of the signals.

The overall signal level, which was found to be comparable across recordings, crucially depends on the power of the laser, viral expression, and intrinsic properties of the calcium indicator. Signal-to-noise ratio (SNR) of the detected transients, in turn, depends on the overall signal level, and may be compromised by shot noise of the photomultipliers at low fluorescence intensities. Motion artefacts, by artificially varying the baseline of the signals, may also contribute to signal degradation. The SNR for different identified neurons significantly varied due to variability of GCaMP6f expression levels between neurons and different positions of neurons with respect to the focal plane of the microscope. As one of the ultimate goals of the analysis of any calcium recording is the inference of spiking activity, one may define SNR of the trace as sufficient, if faithful reconstruction of spiking activity from a given trace is feasible. We used the recently developed Online Active Set method to Infer Spikes (OASIS)^12^ to fit calcium transients and extract decay time parameters which were in the range of 200–600 ms for most of the neurons (Fig. 2, *Decay time (s)*), consistent with what was reported for GCaMP6f for spiking at 1–100 Hz frequency (see e.g. Fig. 1f in^2^). This shows that the majority of the identified neurons in the recordings have a sufficient SNR to allow for extraction of spiking parameters.

Local stability of the recordings (stability within a given recording) was estimated as the ratio between median signal of all neurons in the first and second half of the recording (2^nd^-to-1^st^ half median ratio, and expressed as percentage (for a recording of 9000 frames or 5 min), where 100% indicates that the median signal of the whole neuronal population remained stable and values higher (lower) than 100% indicate an increase (decrease) of the median signal during the recording. For longer or shorter recordings this measure was adjusted accordingly, to make it quasi-independent of the recording length. The ratio of the median signal of the calcium imaging dataset is 100 + -5% (95% C.I.) for all recordings, attesting high local stability (see Fig. 2, Ratio 2^nd^/ 1^st^ half (%)). This suggests that photobleaching of the calcium indicator was negligible during recordings.

### FOV-stability and alignment

Relative shifts of the FOV between recording sessions may occur due to imperfect placement of the animal in the same position or due to slow drift of the brain with respect to the implanted optical window. To quantify the alignment of the field of view of all recordings for a given animal, we implemented a *Similarity index*, which is the result of the comparison of two FOVs with an image-hashing algorithm (ImageHash). This index was used to estimate the ranges of recordings that are well-aligned and thus recommended for the analysis, as they allow for the identity of single neurons to be followed across recordings. We show the *Similarity index* in Supplementary Table 1 as a heatmap that represents the quality of the alignment of any pair of recordings for a given animal.

### Motion

Animal motion was monitored online via a rotary optical encoder mounted on a treadmill to determine periods of motion and annotate datasets. Abovementioned meta-data files contain the “quiet periods” column that includes periods when no movement of the animal occurred to ensure the analysis is restricted to periods of minimal motion artefacts. The choice of these periods was verified by analysis of x- and y-displacements as a proxy of the recording stability (Supplementary Figure 1).

### Electrophysiology

**Fig. 3:**
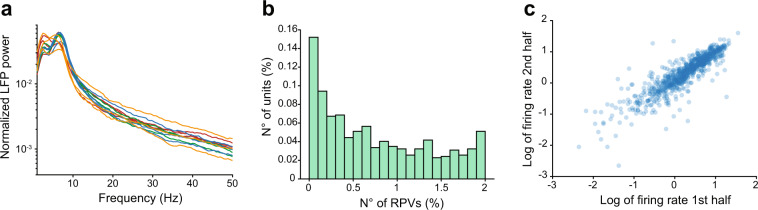
Validation of electrophysiological recordings. (**a**) LFP power spectral density in awake period, different colors indicate different animals. (**b**) Number of refractory period violations in SUA data. (**c**) Correlation between the firing rates in the first and the second half of the data.

To assess the quality of the LFP data and its robustness across different mice, we verified that all recordings from awake mice contained the well-characterized peak in the theta frequency range (6–12 Hz) of the LFP power spectral density (PSD)^13^. To this aim, we quantified the PSD from the recording site located in the stratum pyramidale of the hippocampus and visually confirmed that all PSDs contained a peak in the 6–12 Hz frequency range (Fig. 3a).

To assess the quality of the SUA data, we quantified the number of refractory period violations (RPVs; i.e. the number of action potentials that occurred within less than 2 ms from each other). Of the 743 single units, 478 and 715 had a proportion of RPVs that was smaller than 1% and 2%, respectively (Fig. 3b). Further, we found that there was a high correlation between the estimated firing rate in the first and second half of the recording during wakefulness (0.84 and 0.90, Pearson and Spearman correlation coefficients, respectively) (Fig. 3c). Taken together, these data indicate that SUA activity comes from well isolated neurons, and that the estimated firing rates are robust.

### Pupillometry

**Fig. 4:**
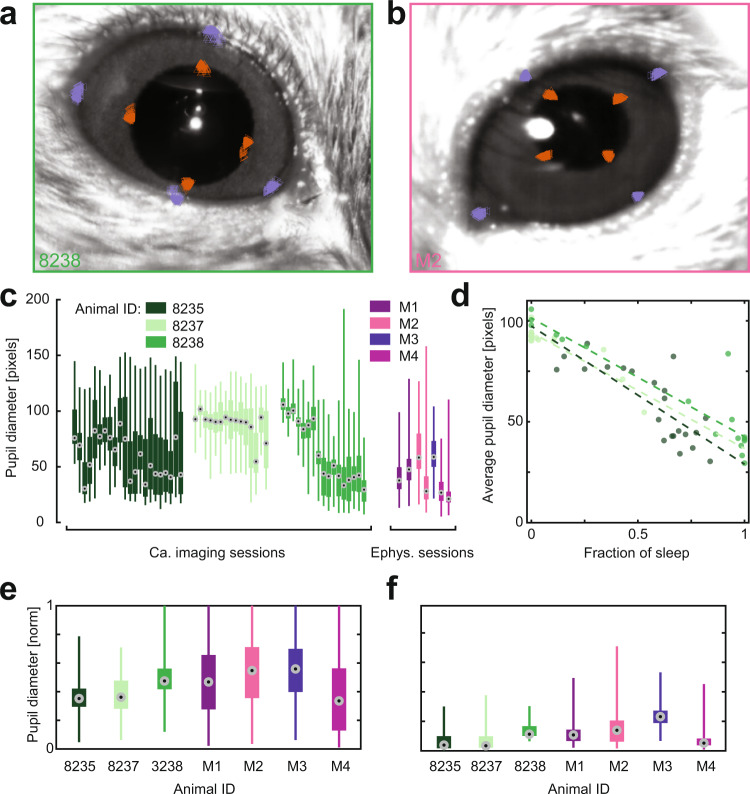
Tracking of eye features and dynamics in pupil size. (**a**) Example of feature tracking during calcium imaging experiments (upper, lower, left, and right edges of the pupil (orange) and eyelid (magenta), n = 100 samples). Images are the mean of 100 subsequent frames. (**b**) Same as (**a**), but for extracellular electrophysiological recordings. (**c**) Raw pupil diameter across sessions, color-coded by animal and session type. (**d**) Average pupil diameter in pixels in each session as a function of the fraction of sleep periods in this respective session. (**e**) Maximum-normalized pupil size for each animal during epochs classified as awake. (**f**) Same as (**e**), but for epochs classified as sleep. The box plots represent the distribution of a parameter in the range between the 1^st^ (Q1) and 3^rd^ (Q3) quartile, the central line is a median (2^nd^ quartile, Q2), while whiskers defined as minimum and maximum values, respectively.

As the first step, pupil and eyelid recordings and feasibility of their analysis were verified by visual inspection, superimposing the features extracted by the DeepLabCut algorithm^11^ with the underlying video (Fig. 4a,b). Furthermore, as the algorithm provides a certainty measure (between 0 and 1) for each feature in each frame, samples with low certainty (**≤**0.5) were linearly interpolated with their next neighboring sample, which had a certainty above 0.5. Based on this data, we subsequently calculated the pupil size and the magnitude of eye opening for each animal. In absolute terms, we observed variability for pupil diameter across experiments, animals, and sessions (Fig. 4c). This variability is expected for various reasons: first, in contrast to electrophysiological recordings, which were performed in a dimly illuminated room, calcium imaging was carried out in darkness with infrared illumination of the pupil (in order to minimize optical noise). Due to the residual ambient light, the pupil was more constricted for animals that underwent electrophysiological recordings as compared to animals used for calcium imaging experiments (68.5 ± 31.8 vs 44.8 ± 22.6 pixels). Second, neither the camera nor the animals were fixed in exactly the same spot during different recording sessions, and manual placement of the camera with respect to the animal in order to focus the image is another source of potential variability. Finally, pupillometry data accompanying electrophysiological recordings resulted from relatively few, but long recording sessions (7 sessions, 322 min on average), while calcium data (and hence pupil recordings) were obtained in multiple shorter sessions (54 sessions, 6.6 min on average) spread over several days. The short duration of pupillometry recordings during calcium imaging sessions adds a further biological source of variability: As the sessions are relatively short, the average pupil size is more prone to the exact ratio of sleep and wake periods (Fig. 4d). This effect is more likely to be cancelled out in the longer sessions of electrophysiological recordings. It is important to note that the pupil size is – besides sleeping and awake states - inherently determined by many contributing sources, including external ones such as object patterns or luminance, and internal ones such as attention, arousal, and even the age of the animal^14–16^. To account for these differences and enable comparable pupil dynamics across sessions and animals, pupil size is typically normalized to the maximum pupil dilation of a given experimental session. By normalizing the pupil data, and by further splitting them into epochs which subsequently were classified as sleeping/waking periods (see^1^), we found the pupil diameter to be comparable across animals, and even across experiments in calcium imaging and electrophysiological recordings in awake (Fig. 4e) and sleep states (Fig. 4f).

## Usage Notes

Raw calcium imaging data requires registration for alignment and motion correction and extraction of neuronal calcium signals with pipelines such as CaImAn^17^ or Suite2p^4,18^. The pre-processing is also an efficient way to compress the data since the algorithms capture biologically relevant correlated signal intensity changes, encoded in associated ROIs. A typical 5-min raw recording occupies 4.4 Gb, while the processed data occupies up to 20-fold less storage space. To infer spiking activity from calcium transients, several algorithms are available: CASCADE^19^, OASIS^12^, Peeling^20^, MLSpike^21^, and Fast Nonconvex Deconvolution^22^. For electrophysiological data, a variety of spike sorting algorithms are available^23^.

**Fig. 5:**
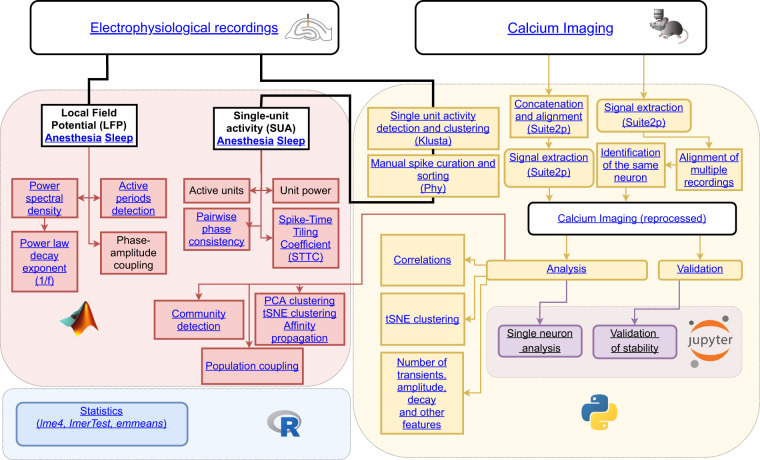
Interactive structure of the analysis pipeline for electrophysiological and calcium imaging data [Interactive version].

As a quick start, we recommend to use the already Suite2p-reprocessed calcium imaging files with *.npy format. While, for electrophysiological recordings, “spike matrices” with *.mat format obtained with *klusta* and *phy* can be readily used. The overall size of reprocessed datasets is smaller and, as discussed in the validation section, they already capture most of the relevant biological signals. An interactive chart in Fig. 5 illustrates a workflow for the data reprocessing and extraction of basic features on the population level developed in^1^. Calcium imaging datasets can be reprocessed in a recording-by-recording manner where one *.npy file with calcium traces corresponds to one *.tif file, or by alignment and concatenation of the recordings with the same field of view. In this case, one *.npy file contains several *.tif recordings merged after the alignment. In the first case, to identify the same neurons across recordings, one may refer to the *Alignment of multiple recordings* and *Identification of the same neuron algorithms*, Fig. 5). Alternatively, the algorithm suggested in Sheintuch *et al*.^24^ can be applied. In the latter case, this additional step for the identification of the same neurons across recordings is not necessary. However, discontinuity on the concatenated traces may occur due to recording-by-recording variation of the laser power, focal drift, or physiological changes as observed in the “Transition State” dataset. Therefore, even if the concatenation procedure is used, it is still recommended to split the concatenated trace into periods that correspond to single recordings rather than analyzing it as a whole.

## Supplementary information

Supplementary Information

## Data Availability

For the calcium trace extraction, suite2p v0.9.3 was used. Suite2p input parameters are specified in the form of *ops.npy* files. Technical validation scripts are available in “validation” folder in^10^. The analysis code, summarized in the interactive Fig. 5, is available in the repositories https://github.com/OpatzLab/HanganuOpatzToolbox (v 1.0.0 Scientific Data release) and https://github.com/mchini/Yang_Chini_et_al (v 1.0.0 Scientific Data release). In addition to the code, Jupyter notebooks are provided to illustrate the calcium imaging validation procedure (Validation_stability.ipynb), and basic analysis approach at the level of individual neurons (analysis_pipeline_for_concatenated_recordings.ipynb).
