# Supplementary material for: Calcium Imaging and Electrophysiology of hippocampal Activity under Anesthesia and natural Sleep in Mice
Source: Sci Data. 2022 Mar 29;9:113. doi: 10.1038/s41597-022-01244-2 (PMC8964694; doi:10.1038/s41597-022-01244-2)
Supplement: Supplementary file 1 — Supplementary Information [file 41597_2022_1244_MOESM1_ESM.pdf]

Supplementary Information

Supplementary Table 1:
Post-alignment of the field-of-view's for datasets page 2

Supplementary Figure 1:
Analysis of x- and y-displacements as a proxy of the recording stability page 5

Supplementary table 1

Mouse ID	Condition	Sessions	Recordings	Duration, frames	Post-Alignment *Similarity index* 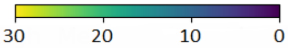
**Natural sleep**					
8235	Natural sleep, awake	2	22	4125 - 19403 / 2.3 - 10.8 min	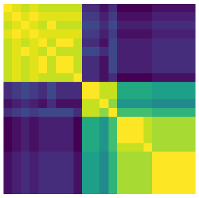
8237	"	2	15	4194 - 26640 / 2.3 - 14.8 min	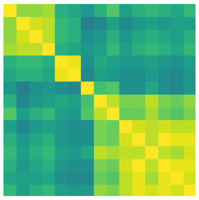
8238	"	2	17	3830 - 25674 / 2.13 - 14.3 min	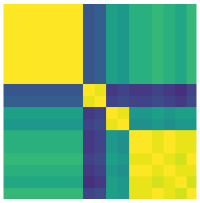
**Anesthesia**					
37527	Keta/Xyl	2	9	9000 / 5 min	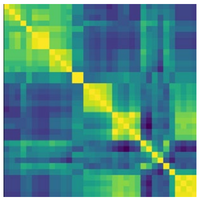
	Isoflurane	2	9	"	
	MMF	2	9	"	
	Awake	2	7	9000 / 5 min 18000 / 10 min	
37528	Keta/Xyl	2	9	"	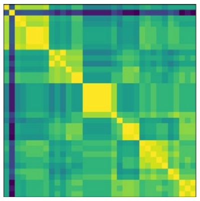
	Isoflurane	2	9	"	
	MMF	2	9	"	
	Awake	2	7	9000 / 5 min 18000 / 10 min	
37529	Keta/Xyl	2	9	"	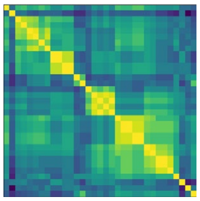
	Isoflurane	2	9		
	MMF	2	9	"	
	Awake	2	6	9000 / 5 min 18000 / 10 min	
37530	Keta/Xyl	2	9	"	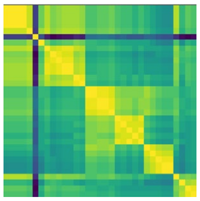
	Isoflurane	2	9	"	
	MMF	2	9	"	
	Awake	2	6	9000 / 5 min 18000 / 10 min	

48	Keta/Xyl	1	4	"	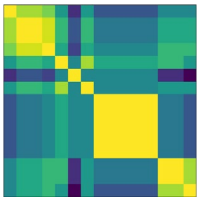
	Isoflurane	1	3	"	
	MMF	1	5	"	
	Awake	1	3	"	
51	Keta/Xyl	1	5	"	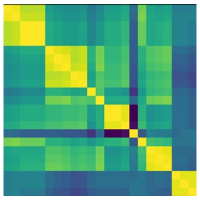
	Isoflurane	1		"	
	MMF	1	5	"	
	Awake / awake quiet	1	5	"	
53	Keta/Xyl	1	5	"	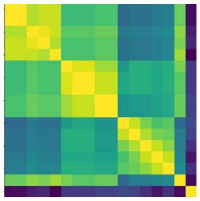
	Isoflurane	1	5	"	
	MMF	1	5	"	
	Awake / awake quiet	1	3	"	
**Transition State**					
8237	Awake, Keta/Xyl, Recovery	1	9	7279 - 18560	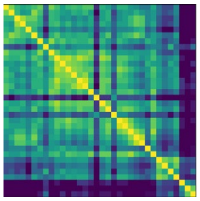
	Awake, Isoflurane, Recovery	1	9	5498 - 14770	
	Awake, MMF, Recovery	1	9	5694 - 16532	
	Awake	1	7	8542 - 18000	
8235	Awake, Keta/Xyl, Recovery	1	9		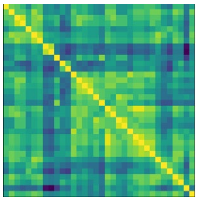
	Awake, Isoflurane, Recovery	1	9	5419 - 9773	
	Awake, MMF, Recovery	1	9	5070 - 10523	
	Awake	1	7	7663 - 19743	
8238	Awake, Keta/Xyl, Recovery	1	9	5284 - 10629	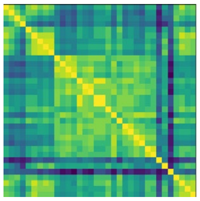
	Awake, Isoflurane, Recovery	1	9	6160 - 33970	
	Awake, MMF, Recovery	1	9	6518 - 12873	
	Awake	1	7	7525 - 12151	
F0	Awake, Keta/Xyl, Recovery	1	9	8573 - 18000	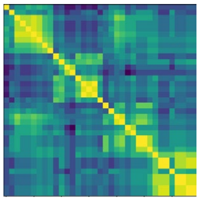
	Awake, Isoflurane, Recovery	1	9	10327 - 18000	
	Awake, MMF, Recovery	1	9	6111 - 18000	
	Awake	1	8	8919 - 18000	
M3	Awake, Keta/Xyl, Recovery	1	9	8538 - 18000	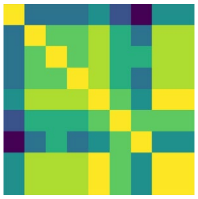

M0	Awake, Isoflurane, Recovery	1	9	5702 - 18000	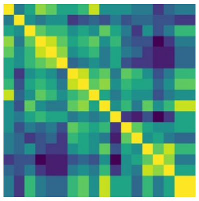
	Awake, MMF, Recovery	1	9	7217 - 18000	
F1	Awake, Keta/Xyl, Recovery	1	9	9112 - 29202	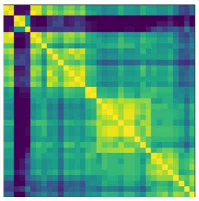
	Awake, Isoflurane, Recovery	1	9	8214 - 18000	
	Awake, MMF, Recovery	1	9	8437 - 18000	
	Awake	1	8	8542 - 18000	

Supplementary Table 1. Post-alignment of the field-of-view's for datasets; quantification by the *Similarity Index*. Yellow represents the highest quality of the alignment (*Similarity index* = 30), while dark blue the lowest (*Similarity index* = 0, not possible or hard to align). The order of the recordings in post-alignment heatmap is the same as in meta-data files.

Supplementary figure 1

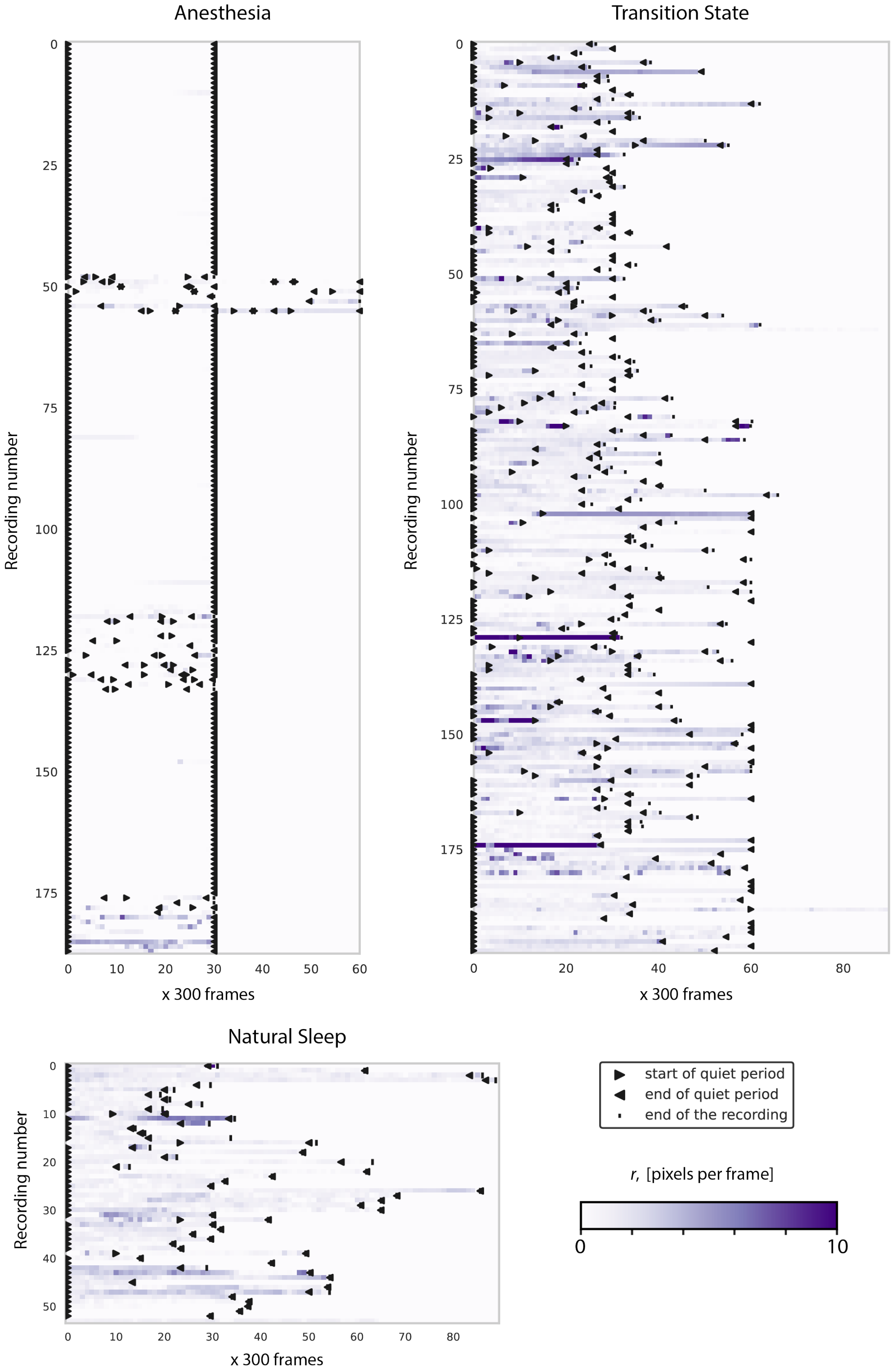

Supplementary figure 1. Analysis of x- and y-displacements as a proxy of the recording stability. Displacements over two dimensions are jointly quantified as an $r = \sqrt{x^2 + y^2}$ parameter averaged over 300 frames and expressed in [pixels per frame].
